# Training health care providers in abortion and sexual and reproductive rights: challenges and recommendations

**DOI:** 10.3389/fmed.2026.1782347

**Published:** 2026-06-26

**Authors:** Henry Castro, Adela Montero, Claudio López-Labarca, Heidy Kaune, Mercedes Carrasco-Portiño, Leonardo Reyes-Torres, Lidia Casas, Alejandra Ramm

**Affiliations:** 1 Departamento de Obstetricia y Ginecología, Facultad de Medicina, Universidad de Concepción, Concepción, Chile; 2 Centro de Medicina Reproductiva y Desarrollo Integral de la Adolescencia (CEMERA), Facultad de Medicina, Universidad de Chile, Santiago, Chile; 3 Departamento de Obstetricia y Puericultura, Facultad de Ciencias y Salud, Universidad de Atacama, Copiapó, Chile; 4 Programa de Ética y Políticas Públicas en Reproducción Humana, Facultad de Medicina, Universidad Diego Portales, Santiago, Chile; 5 Departamento de Obstetricia y Puericultura, Facultad de Medicina, Universidad de Concepción, Concepción, Chile; 6 Centro de Derechos Humanos, Facultad de Derecho, Universidad Diego Portales, Santiago, Chile; 7 Escuela de Sociología, Universidad de Valparaíso, Valparaíso, Chile

**Keywords:** abortion training, gender equity, health workforce education, Latin America, sexual and reproductive health

## Abstract

Persistent gaps remain in the training of physicians and midwives in sexual and reproductive health in Chile, despite overall advances in the field. These shortcomings affect equitable access to care and contribute to stigma surrounding sexual and reproductive rights. A multicenter study involving over 2,500 students and 57 professors from Chilean universities identified uneven curriculum implementation and disparities between secular and religious institutions. This policy brief proposes evidence-based interventions, including comprehensive sexuality education; integration of gender and human rights perspectives; explicit inclusion of abortion, conscientious objection, and confidentiality; incorporation of these topics into national assessment requirements; and strengthening faculty competencies. The proposed actions aim to improve healthcare quality and align with standards set by the WHO, FIGO, and other international organizations.

## 1 Introduction

In Chile, sexual and reproductive health has made significant strides ever since the Chilean government established a Midwifery School in 1834, most notably with the adoption in 2017 of legislation regulating voluntary pregnancy termination on three grounds (1). These gains, however, coexist with enduring structural gaps, including educational and institutional barriers –such as conscientious objection– that weaken sexual and reproductive rights and restrict equal, timely access to voluntary pregnancy termination (2). Several studies have shown that such barriers can delay or prevent access to safe abortion and expose women to unsafe procedures, fragmented care, and increased morbidity and mortality. This is exacerbated whenever disparities based on age, ethnicity, and socio-economic or migration status converge to create intersectional scenarios that amplify the negative effects (3). In addition to good laws, effective access to safe abortion requires technically, humanely, and ethically competent providers, but subpar formal education remains a key structural barrier (4). If abortion is legal but not covered in school, graduates will lack clinical and regulatory tools, which leads to uncertainty, institutional barriers, undue deterrents, and delays in care (5). Schools and other key actors in health system transformation that leave abortion off the syllabus help obscure its essential nature, reproduce the associated stigma, and bolster a health care model that restricts sexual and reproductive health rights (6).

Internationally, the World Health Organization (WHO), the International Federation of Gynecology and Obstetrics (FIGO), the Royal College of Obstetricians and Gynecologists (RCOG) (7), the American College of Obstetricians and Gynecologists (ACOG), the Society of Obstetricians and Gynecologists of Canada (SOGC), and the International Federation of Medical Students’ Associations (IFMSA) all advocate abortion training in medical graduate and undergraduate programs. These organizations have urged schools to provide training based on clinical, legal, ethical, and communication skills (8).

This policy paper addresses sexual and reproductive health and rights gaps in provider training. In our 2023 Multicentric Study on Perceptions and Attitudes of Medical and Obstetrics Students and Teachers on Abortion in Chile (9), we surveyed over 2,500 students countrywide and conducted interviews with 57 faculty members from both secular and faith-based schools. This paper draws from the study’s findings and from domestic and international evidence. The recommendations presented here are intended to improve training in sexual and reproductive health and rights, especially abortion, standardize it across graduate and undergraduate health programs, and advance science-based education based on a compassionate, comprehensive, equitable, rights-centered approach. In sum, this brief seeks to improve access as well as the timeliness and quality of sexual and reproductive health care, with emphasis on abortion. It is intended for technical and higher education institutions offering health care programs as well as for the relevant policymakers.

## 2 Analysis from the evidence

### 2.1 Suboptimal sex education

As taught in Chilean schools, sex education is noted for its narrow, checkered approach. While public curricular guidance exists, schools choose how to implement it, often focusing on the biology of reproduction while neglecting issues of emotions, consent, and diversity. The consequences translate into individual and social issues such as mounting rates of sexually transmitted infections (STI), early sexual debut and associated risky behavior, and stigmatization and misinformation about issues such as pleasure and sexual and gender diversity (10, 11). In Latin America, research shows that school programs tend to focus on risk and prevention over rights and well-being, which reproduces gender stereotypes and overlooks the emotional and affective dimensions (12). As the preliminary results of our Multicentric Study confirm, most student respondents report that sex education received in school was mostly focused on condom use, anatomy, and STI prevention. A total of 38.4 percent of students at faith-based universities gave a failing grade to the sexual and reproductive health education received, in contrast to 17 percent at non-confessional universities. Some 32.3 percent of medical students evaluated sex education poorly, versus 8.7 percent of midwifery students. These variations are statistically significant.

### 2.2 Abortion training

Health program student attitudes and perceptions have evolved positively in recent decades. General trends show them to be more amenable to the provision of abortion services than the general population, but they still consider their training insufficient (8, 13, 14). In the preliminary Multicentric Study results, over 85 percent of student respondents pointed at training gaps and stated a willingness to acquire more comprehensive abortion care skills. In addition, more than 80 percent considered abortion to be a right and part and parcel of standard health care services. This mindset is in line with international findings that report a generational shift toward acceptance of abortion as an essential component of the right to choose (8).

### 2.3 The role of teachers and the hidden curriculum

In the absence of a national educational policy that explicitly integrates sexual and reproductive health and rights–including abortion–the coverage of these topics remains contingent on individual teacher judgment and institutional value systems. Teachers may consider these topics irrelevant or inappropriate, or as advanced skills best reserved for specialists (15). The preliminary qualitative results of the Multicentric Study describe a hidden curriculum whereby teachers will adapt content based on context and personal values, and in faith-based universities, even self-censor. These issues are compounded by restrictions explicit or implicit in stated school institutional values and beliefs. These do not necessarily imply the wholesale omission of contents related to the law, but rather the use of more generic, oblique approaches contingent on personal standpoints. Yet, the Multicentric Study also found that while sexual and reproductive health instruction lacks a unified approach, clinical practice provides a unique chance to teach ethical and attitudinal skills that reinforce empathy and responsibility. The literature has documented that faith-based institutions tend to restrict the abortion debate, which creates training disparities among graduates (16).

### 2.4 Low prevalence of conscientious objection and peer discouragement

Global studies show intent to object to abortion at 5–11 percent. Studies conducted in Chile confirm that conscientious objection stands at below 10 percent across grounds, including those outside the scope of the voluntary pregnancy termination law (17). Preliminary results from the Multicentric Study show that only 6 percent of medical students would never perform an abortion. Among midwifery respondents, intent to object decreased as the program progressed, especially for reasons other than value-based. In medical schools, although intent remained constant, the motives for claiming objector status changed, with fewer non-value-based reasons. This is consistent with research showing that clinical exposure improves ethical empathy and reduces objection based on fear and prejudice (18). This relates to the notion of the pseudo-objector who claims the status for fear of social stigma or disapproval by family or colleagues rather than for ethical, moral, or faith-based reasons. In the Multicentric Study, over 85 percent of respondents disagreed with practices such as discouraging women from accessing abortions or persuading peers not to perform them. This is in line with research showing a tendency to deem conscientious objection as an impediment to equal access (8, 19). Qualitative Multicentric Study results further show that the quality of teaching on conscientious objection is heterogeneous and greatly depends on institutional values and faculty views. Some faith-based universities teach conscientious objection as a right, while some non-confessional universities address the issue only in passing. Research in similar contexts shows that fragmented teaching practices restrict development of clinical and ethical competencies on abortion (14, 15).

### 2.5 Confidentiality and doctor-patient privilege

In the preliminary Multicentric Study results, over 80 percent of respondents agreed with the need for confidentiality in abortion contexts and that reporting flouts the duty of care. Interviews with teachers revealed a clear, formal approach to doctor-patient confidentiality in relation to HIV or teenage patients but not to voluntary pregnancy termination, demonstrating unawareness of domestic and international regulations. International studies confirm this point (8). Equivocation in this respect has been documented elsewhere as undermining trust in services and reinforcing the abortion stigma and also shows that subpar training on confidentiality in abortion cases can lead to uncertainty and to evasive clinical practices (5).

## 3 Actionable recommendations

Implement cross-disciplinary, comprehensive courses that include sex and gender, diversity, pleasure, and self-awareness for all graduate and undergraduate students in health-related programs per FIGO/IFMSA/World Association of Trainees in Obstetrics & Gynecology (WATOG) recommendations (Joint statement of support for the inclusion of contraception and abortion in sexual and reproductive health and wellbeing education for all medical students).

Build a gendered, rights-based sexual and reproductive health approach into the curricula of all graduate and undergraduate health training programs.

Include content on abortion, conscientious objection, and patient confidentiality into all health degree and medical residency programs, based on an ethical, legal, and human rights approach.

Set minimum sexual and reproductive health training standards for all schools offering health degrees and medical residency programs.

Include sexual and reproductive health content, including on abortion and the voluntary pregnancy termination law, in the requirements for assessment and accreditation of health degrees and residency programs.

Train and update concerned faculty on key sexual and reproductive health and rights content, including on abortion, conscientious objection and patient confidentiality, through competency-based programs ensuring unbiased, ethical, rights-based instruction.

## 4 Conclusion

Normative advances do not automatically translate into timely and high-quality access to care in the absence of adequately trained professionals. This underscores the vital role of professional education in the effective implementation of sexual and reproductive health legislation. Related care depends not only on technical competencies, but also on solid and coherent ethical and communication training. In contexts where clear training standards are lacking, disparities in professional preparation reflect the predominance of institutional values over individuals’ right to health. In this sense, strengthening training standards does not constitute an ideological choice, but rather an essential requirement to ensure rights. Professional education should therefore be understood as a facilitator–rather than a structural barrier–to effective access to legally recognized sexual and reproductive health services in each country.
